# HIV DNA Vaccine: Stepwise Improvements Make a Difference

**DOI:** 10.3390/vaccines2020354

**Published:** 2014-05-14

**Authors:** Barbara K. Felber, Antonio Valentin, Margherita Rosati, Cristina Bergamaschi, George N. Pavlakis

**Affiliations:** 1 Human Retrovirus Pathogenesis Section, Vaccine Branch, Center for Cancer Research, National Cancer Institute at Frederick, P.O. Box B, Frederick, MD 21702, USA; E-Mail: cristina.bergamaschi@nih.gov; 2 Human Retrovirus Section, Vaccine Branch, Center for Cancer Research, National Cancer Institute at Frederick, P.O. Box B, Frederick, MD 21702, USA; E-Mails: antonio.valentin@nih.gov (A.V.); margherita.rosati@nih.gov (M.R.); george.pavlakis@nih.gov (G.N.P.)

**Keywords:** macaque, cytokine, plasmid, electroporation, immunogenicity, vaccination, DNA delivery, DNA expression

## Abstract

Inefficient DNA delivery methods and low expression of plasmid DNA have been major obstacles for the use of plasmid DNA as vaccine for HIV/AIDS. This review describes successful efforts to improve DNA vaccine methodology over the past ~30 years. DNA vaccination, either alone or in combination with other methods, has the potential to be a rapid, safe, and effective vaccine platform against AIDS. Recent clinical trials suggest the feasibility of its translation to the clinic.

## 1. Introduction

Different vaccine approaches, including the use of plasmid DNA, recombinant viral vectors, protein or peptides and combination thereof in prime-boost regimens are being pursued as potential vaccines against HIV/AIDS. More than 30 trials are currently being conducted to evaluate the immunogenicity of promising HIV vaccine candidates. Four efficacy clinical trials have been completed: (i) gp120 (VaxGen) [1,2,3,4]; (ii) recombinant Ad5 (STEP) [5,6,7]; (iii) DNA prime-recombinant Ad5 boost (HVTN 505) [8]; (IV) combination of recombinant Canarypox ALVAC^®^-HIV (vCP1521; containing Gag, PR and Env) with gp120 Env protein (AIDSVAX^®^ B/E) [9]. Although the first three trials failed to show efficacy, the latter resulted in modest statistically significant protection from infection in the RV144 vaccine trial conducted in Thailand [9]. The limited efficacy and the fact that the vaccine-induced responses waned over time indicate that improved vaccine designs are needed to achieve long-lasting cross-clade immune responses able to prevent and contain infection. Importantly, the RV144 trial revealed a critical role of humoral responses in preventing infection. Anti-Env IgG antibodies targeting the V1V2 region correlated with protection from infection [10,11,12,13,14]. No vaccine-induced virus control was observed in the individuals who became infected, indicating that this vaccine failed to elicit efficient immune responses able to control viremia during the chronic phase of infection. Ideally, an effective vaccine against HIV should provide durable cross-clade humoral immune responses at the portal of entry and arm the cellular immune system with cytotoxic effector memory cells able to contain or eliminate any break-through infection. Therefore, there is urgent need to identify more efficacious vaccine regimens. The use of DNA as vaccine platform is promising due to its simplicity, scalability, and possibility of repeated applications due to the lack of immunity against the vector (for reviews see [15,16,17]). To date, three DNA vaccines for animal use have been approved: (i) against equine West Nile Virus infection in horses [18,19]; (ii) a therapeutic cancer vaccine against melanoma in dogs [20]; and (iii) a vaccine against infectious hematopoietic necrosis virus (IHNV) in salmon [21,22,23]. In the HIV vaccine field, few trials have used DNA as the single vaccine modality due to its relatively low immunogenicity in primates, but DNA vaccination was shown to protect macaques from chronic viremia [24,25,26]. Yet, DNA vaccination methods that optimize immunogen expression and delivery have made great progress in improving immune responses, and it was further shown that DNA vaccines induce long-lasting immunity against HIV/SIV in non-human primates [24,27,28,29]. 

## 2. Regulated HIV *gag* and *env* Expression

The HIV Gag and Env proteins represent key targets for vaccine development. However, from the onset of vaccine development in the mid 80s, HIV DNA as vaccine regimen was found to be poorly immunogenic. Several obstacles needed to be overcome to make DNA a contender in the race of developing an HIV vaccine, including levels of antigen expression and DNA delivery. We now know that the level of antigen expression is critical to induce a potent immune response, but in the past many labs failed to get good expression of HIV Gag and Env, resulting in very poor immunogenicity in animal models. In retrospect, the limited understanding of the rules for efficient gene expression had a serious negative impact resulting in suboptimal levels of antigen production and, subsequently, modest immunogenicity. The reason for this failure is based on the fact, that expression of HIV *gag* and *env* is regulated by the viral Rev protein (for reviews see [30,31,32,33] and references therein]. Rev is essential for the export of the unspliced and partially spliced mRNAs [34] encoding the structural viral proteins, and mediates production of infectious virions. Rev-minus virus mutants are unable to replicate [35,36], whereas trans-complementation of the Rev-minus HIV with Rev restored expression of *gag* and *env* mRNA as well as virus production, demonstrating the essential role of this regulatory factor. Rev interacts with the *cis*-acting RNA recognition motif termed Rev Responsive Element (RRE) [37,38], a highly structured RNA element embedded within the *env* coding region present only in the unspliced and partially spliced HIV mRNAs, and promotes their export and expression. The discovery of the HIV Rev mechanism of function opened up new opportunities to understand nucleo-cytoplasmic transport, and posttranscriptional regulation in general, of both viral and cellular mRNAs (reviewed in [30]).

Dissection of the underlying mechanism led to the identification of several regions embedded within the intronic regions in the *gag*, *pol* and *env* coding sequences termed INS (instability sequences) or CRS (*cis*-acting repressive signals) that have a *cis*-acting negative effect on viral mRNA expression [37,39,40,41,42,43,44] and affect stability, export and expression of this subset of viral mRNAs (see below). Together, the combination of Rev *in trans* and its RNA interaction site RRE *in cis* corrects the defect caused by these negative-acting sequences resulting in efficient Gag/Pol and Env expression and virus production. In conclusion, recognition of this basic regulatory mechanism mediated by the viral Rev protein proved to be the key to achieve efficient expression by HIV *gag* and *env* encoding plasmid DNAs.

## 3. Method of RNA/Codon Optimization to Circumvent the Poor Expression of HIV *gag*/*pol* and *env*

The discovery of the role of Rev in the expression of HIV *gag*/*pol* and *env* provided critical information on how to overcome the poor expression from recombinant vectors containing sub-genomic HIV regions. Yet, this arrangement did not lead to high levels of expression in mouse cells, because the Rev protein does not work efficiently in these cells. This limitation made the study of several recombinant HIV vaccines very challenging or impossible in mice and rabbits, critical models for studying immunogenicity.

The question then arose whether alternative methods could be found to make the HIV *gag* and *env* expression independent of the viral regulatory factor Rev. The initial identification of the negative-acting INS sequences within the coding sequence of *gag* mRNA that were inhibiting expression and affecting the stability of mRNA [40,41], even when placed outside the translated region, suggested they were acting at the level of the mRNA and not during translation at the ribosome. These facts supported the model in which INS/CRS elements provide interaction sites for cellular factors that negatively affect the fate of these transcripts in the nuclear compartment, which consequently affects their fate in the cytoplasm inhibiting translation. This down-regulatory effect can be counteracted by the presence of the HIV posttranscriptional regulatory systems (Rev-RRE). Alternatively, Rev-RRE can be replaced by other RNA export systems such as the retroviral *cis*-acting transport elements CTE or RTE, acting via the cellular NXF1 and RMB15 proteins (reviewed in [30] and references therein). A third method developed by us [40,41] was the removal of the negative-acting RNA instability signals (INS) through RNA/codon optimization. 

This alternative method, RNA/codon optimization, which changes the nucleotide sequence without altering the coding potential of the mRNA, was initially achieved by altering the nucleotide composition within eight INS regions in the *gag* mRNA. Changes in 81 of the 1500 nt in the *gag* gene (changing the sequence of 69 of 500 codons in *gag*) without altering the produced protein led to a profound >100-fold increase in Gag protein expression in the absence of Rev/RRE [39,40,41]. It is now apparent that INS elements interact with many different factors and thus they have diverse nucleotide sequences. Some INS elements contain classical AU-rich elements with the signature motif AUUUA also found in the 3'UTR of many cytokine and other mRNAs. Such elements are responsible for the posttranscriptional control of many cellular genes. The INS elements exert their function independent of splicing, even when placed 3' of the coding sequence. A proof that INS elements act at the level of mRNA in the nucleus is provided by the fact that INS have no negative effect on expression from recombinant poxvirus vectors (MVA, ALVAC), which produce mRNAs that are confined to the cytoplasm, and therefore “escape” the regulated nuclear export process. In contrast, expression of INS-containing mRNAs from recombinant Adenovirus and Herpes-based vectors is severely affected, because these viruses go to the nucleus and depend on the nuclear export machinery for their expression.

Thus, RNA/codon optimization inactivates the RNA-embedded inhibitory signals (INS) and results in high level Rev-independent production of Gag, Pol and Env (for detailed references see [30]). The discovery of INS/CRS and their effective elimination by RNA optimization have important practical application in the use of such RNA optimized HIV genes in DNA plasmids or recombinant viral vectors currently used in many vaccine studies in monkeys and humans. This general method of RNA/codon optimization is a commonly applied technology for gene transfer studies; it is used successfully for the optimization of numerous viral and cellular mRNAs and results in great gains in mRNA stability, transport and expression. Importantly, most of the candidate AIDS vaccines moving towards the clinic incorporate RNA/codon optimization to achieve efficient antigen expression. 

## 4. Methods to Improve HIV/SIV DNA Vaccine Regimen

**Figure 1:**
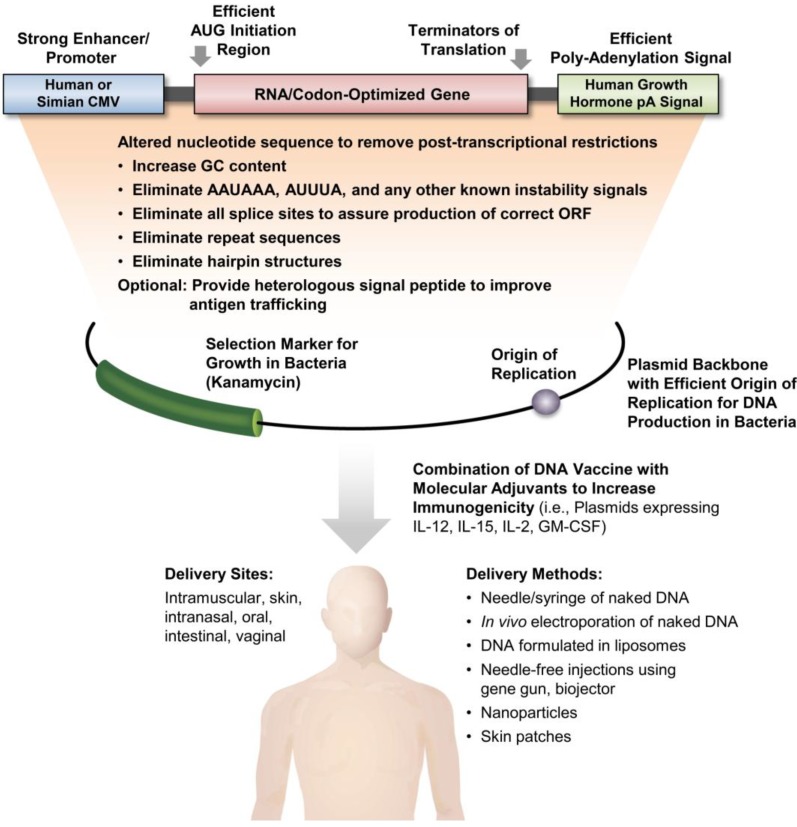
Optimization of DNA expression and delivery: from bench to bedside. Several steps are necessary to improve the efficiency of DNA as vaccine including RNA/codon optimization of the gene, the use of optimized expression vectors, and combinations of DNA vaccine with molecular adjuvants to increase immunogenicity and using different delivery methods and sites.

Several steps are critical to maximize the efficacy of an HIV DNA vaccine regimen (Figure 1), including optimization of plasmid DNA, optimization of immunogen, DNA delivery method, and the inclusion of molecular adjuvants. (i) To maximize antigen production from plasmid DNA, the RNA/codon optimized genes (see above) are inserted into expression vectors which typically use the human CMV enhancer/promoter and provide a potent initiation of translation signal such as the Kozak sequence (5'-gccgccaccATG(G)-3') or the HIV-1 tat sequence (5'-aagaaATG(G)-3') to initiate translation of the gene of interest and the bovine growth hormone (BGH) polyadenylation signal in a plasmid backbone optimized for replication in bacteria, which may also contain antibiotic genes as selection, typically the kanamycin gene. (ii) A second parameter to improve immunogenicity has also been extensively explored, namely the modification of the natural immunogen. One of the main features of DNA vaccines is versatility and ease of rapid alterations of the expressed immunogen. Although initially the focus was to produce authentic viral proteins as immunogens, it was discovered that modification of the produced immunogen could have advantages as a vaccine. In addition to modifications resulting in increased expression, deletions and fusions of immunogens may lead to improved secretion, rapid degradation or transport to different cellular compartments with the ultimate goal of enhancing immunogenicity. Many such variations have been applied taking advantage of the easy methodology provided by genetic engineering. For HIV/SIV antigens, several methods are employed to improve secretion using signal peptides such as tissue plasminogen activator (tPa) [45,46], or granulocyte-macrophage colony-stimulating factor (GM-CSF) [47]; addition of IgE leader to improve expression [48]; embedding the antigen within LAMP to target the fusion protein to the major histocompatibility complex type II (MHC II) processing compartment [49,50,51], fusion to MCP-3 to target immunogens to antigen-presenting cells [26,49], and fusion to signals promoting proteasomal degradation and presentation by the MHC class I molecules such as ubiquitin [52,53,54] or beta-catenin [26]. (iii) An important milestone in making DNA an attractive vaccine vehicle has been the improvement of *in vivo* delivery. Naked DNA is picked up poorly by primary cells and its expression is minimal. To improve delivery to the nucleus, several methods have been developed including intramuscular DNA delivery by *in vivo* electroporation (IM/EP) ([55,56,57,58,59,60,61] reviewed in [62,63,64,65]); skin or intradermal electroporation [66,67,68,69,70,71,72,73], skin patches [74], liposome delivery with Vaxfectin^®^ [75,76], DNA formulated in liposomes [77]; gene gun [78] or biojector [79,80,81]. (iv) Fourth, different strategies to increase the immunogenicity of HIV DNA vaccination in macaques are being pursued, including combination of improved DNA vectors and cytokine DNAs, *i.e.*, IL-12 [82,83,84,85,86,87,88], IL-15 [89,90,91]; IL-2 [89,92,93,94]; GM-CSF [95,96,97,98], chemokines such as RANTES [99] and costimulatory molecules such as CD40L [100,101,102]. (v) In addition to intramuscular and intradermal routes, DNA can also be delivered via the intranasal, oral, intestinal, and vaginal routes [89,92,93,103].

We have been focusing on the use IL-12 DNA as molecular adjuvant together with RNA/codon optimized HIV/SIV DNA vaccines in macaque studies [88,104]. Importantly, inclusion of IL-12 resulted in an increase in both vaccine-induced magnitude and breadth of cellular and humoral immunity, even in combination with the efficient electroporation delivery [60,88,104]. In addition, a recent human trial using HIV *gag* DNA showed that inclusion of IL-12 DNA is advantageous, resulting in both increased frequency of responders and level of Gag-specific immunity [105,106], which is in agreement with the data obtained in macaques. Together, the use of IM/EP delivery and inclusion of IL-12 DNA showed a major improvement for HIV DNA vaccine immunogenicity in humans. Previous vaccine trials indicated that the magnitude of immune responses after DNA vaccination using conventional injection methods is low in humans ([107,108,109,110,111,112,113,114,115,116,117]; reviewed in [118]), similar to the conclusions from macaque studies using the same methodology. Clearly, successive studies showed incremental improvements of vaccine-induced immunity. Findings from the macaque model were shown to translate well into clinical trials [105,106,119], validating the ability of this model to identify improved vaccine candidates. As a result of recent studies, there is a strong and renewed interest in DNA vaccines, due to the accumulating evidence of their increased immunogenicity in humans. Importantly, the robust and effective cellular immunity achieved by optimized DNA is also an important consideration for the expanding field of cancer vaccines. 

## 5. HIV/SIV DNA Vaccine Provide Persistent Immunity

A critical feature of any vaccine is the longevity of the induced immune responses. The RV144 vaccine trial in Thailand [9] elicited immune responses that waned rapidly after the vaccination period, suggesting that the transient nature of the elicited immunity could be at least partially responsible for the limited vaccine efficacy. A first study assessing the longevity of the immune responses elicited by EP-delivered DNA vaccines in non-human primates showed persistence of Env humoral responses over one-year of follow-up [120]. We investigated the durability of both cellular and humoral immune responses elicited by the EP delivered DNA vaccine in macaques [28] and reported that SIV DNA vaccination was able to induce persistent immune responses, which were boosted with each subsequent immunization, even after an extended 90-week rest period, indicating long-lasting vaccine-induced immunological memory. We found remarkable durability of Gag and Env immune responses in DNA EP vaccinated macaques for several years [28,29]. Other DNA delivery methods, such as intradermal EP or use of Vaxfectin^®^ as a cationic lipid-based formulation, also induced potent immunity with impressive durability detectable for 1–2 years in macaques [76,121,122]. Together, these studies demonstrate another important property of DNA vaccines, which is the remarkable durability of the elicited immune responses.

## 6. HIV-1 Diversity and DNA Vaccine

HIV sequence diversity and the presence of potential immunodominant “decoy” epitopes are additional hurdles in the development of an effective AIDS vaccine. The plasticity of HIV allows the virus to escape from the immune system generating an enormous number of variants. Another hurdle has been the observation that immunodominant (ID) epitopes present within HIV proteins may impair the induction of more relevant responses [123,124,125,126,127,128,129,130,131,132,133,134]. These issues need to be taken into consideration for successful vaccine design. Several approaches are being explored, including strategies that use consensus, center-of-tree or ancestral sequences, combination of multiple strains, mosaic immunogens, which are composed of *in silico* recombined natural sequences, immunogens composed of previously identified epitopes, and chimeric molecules expressing a selection of the most conserved epitopes from different clades of HIV ([47,135,136,137,138,139,140,141,142,143,144,145,146,147,148,149,150,151,152,153], reviewed in [154]). 

One of our vaccine approaches focuses on the development of immunogens based on strictly conserved elements (CE) of HIV-1 M group [155,156], which induce immune responses to nearly invariable proteome segments, while excluding responses to variable and potentially immunodominant “decoy” epitopes. We developed a prototype CE DNA vaccine and demonstrated that immunization with this conserved element DNA elicited robust cellular and humoral immune responses against CE, which cannot be achieved by full-length immunogen vaccination in mice and macaques [47,153,157]. We demonstrated that priming with CE DNA and boosting with DNA expressing the full-length immunogen is an effective strategy to maximize responses against Gag, providing a novel concept to increase the magnitude and breadth, including epitopes within highly conserved elements, of vaccine-induced immune responses [153,157]. Thus, inclusion of the CE immunogen as part of an HIV-1 vaccine provides a novel and effective vaccine strategy to avoid eliciting responses against potentially immunodominant decoy epitopes, while focusing the responses to critical elements of the virus. The testing of this novel concept in humans will be pursued in a clinical trial.

## 7. Optimizing Both Arms of the Immune System by DNA & Protein Co-immunization Strategy

Initial efforts for the development of an HIV vaccine showed poor immunogenicity [109], and improved immunogenicity was only found upon using the efficient delivery method of *in vivo* electroporation [105,119,158]. Several vaccines using DNA as a prime in combination with different boosts are also being pursued in clinical trials, including DNA prime-protein boost [114,159], DNA in combination with rMVA [160,161,162,163,164,165] and DNA prime-rAde boost [8]. 

As an alternative strategy, we combined the DNA and protein vaccine modalities in a co-immunization regimen. This strategy is based on two observations: (i) DNA vaccination is able to elicit strong cellular immunity, whereas compared to a protein only vaccine, the antibody responses are lower; and (ii) protein vaccination induces strong antibody responses but less robust cellular immunity. Therefore, it was thought that combining these two vaccine regimens into a co-immunization strategy might be ideal to optimally trigger both arms (humoral and cellular) of the immune system. Based on initial observations, co-immunization with DNA delivered by needle and syringe and inactivated SIV virus particles as the protein source induced antibodies that were higher in magnitude and longevity than either component alone [29]. The co-immunization concept was further tested in mice [166,167], rabbits [167] and macaques [24,29,166]. Collectively, it was found that HIV DNA & protein co-immunization was superior in eliciting humoral immune responses to vaccination with either of the two individual components alone, even when DNA was administered by the more efficient EP method. Importantly, HIV DNA & protein co-immunization vaccine regimen did not decrease the magnitude or alter the specificity of the cellular responses [24,29,166]. In addition to inducing the highest systemic binding and cross-neutralizing antibodies to HIV and SIV Env [29,166], this vaccine regimen also induced the highest Env-specific IgG in saliva [29] and promoted improved dissemination of immune responses to mucosal sites, including rectal fluids [24,29,168]. 

In conclusion, DNA & protein co-delivery in a simple vaccine regimen combines the strength of each vaccine component, resulting in improved magnitude, extended longevity and increased mucosal dissemination of the induced antibodies in macaques. 

## 8. Efficacy of DNA Vaccine in the Macaque Model

Using DNA as the only vaccine component, we and others previously reported the induction of protective immune responses against SIV or SHIV [24,25,26,99,169,170,171,172,173], supporting the effectiveness of SIV DNA vaccines to induce potent T cell responses with proliferative capability and multi-functionality, including cytokine secretion and cytotoxicity. Among several parameters, we found that vaccine induced SIV-specific CD4^+^ cytotoxic T cells contributed to control of viremia [24]. Interestingly, such antigen-specific cytotoxic memory CD4^+^ T cells were also found to contribute to virus control in macaques infected with a truly non-pathogenic live-attenuated SIV [174]. 

Other DNA based vaccination regimens including DNA as prime followed by heterologous boosts, e.g., protein [175,176,177,178]; rNYVac [179,180,181]; rAde [104,182,183,184,185,186,187,188,189,190,191,192,193,194,195]; rHSV [196]; rMVA [87,89,94,95,96,103,197,198,199,200,201,202,203,204] also reported both immunological benefit and reduction of viremia to different extents. Other prime-boost regimens, e.g., recombinant Rubella vectors [205], also showed promising immunological benefit. Taken together, these studies demonstrate that the DNA vaccine platform, known for its potency as T cell vaccine, also contributes to protection from infection.

Interest in using DNA as a vaccine vehicle for HIV is not limited to preventive vaccine but also for potential application as therapeutic vaccine. Using the SIV/macaque model, we demonstrated virological benefit induced by therapeutic DNA vaccination in SIV macaques under anti-retroviral treatment (ART). DNA vaccination administered by needle and syringe via the IM route or by IM/EP elicited potent cellular responses able to greatly reduce virus load upon release from ART, leading to durable control of viremia over many months [90,206]. Because DNA vaccination can be repeatedly administered without development of immunity to the vector, repeated cycles of therapeutic vaccination resulted in a great benefit with a further reduction in viremia. Recent therapeutic trials using DNA vaccines showed improved immunogenicity ([207,208,209,210], for recent review see [211,212]) in agreement with findings of the macaque model. Thus, vaccination with plasmid DNAs has several advantages, including the possibility for repeated administration, and was shown to induce potent, efficacious, and long-lasting immune responses.

## 9. Perspective and Conclusions

DNA vaccine has great advantages in versatility, scalability, safety and relative simplicity of manufacturing. For HIV, research over the past ~30 years has shown that understanding the basic molecular biology of an immunogen is critical to generating efficient expression vectors, which together with improved DNA delivery, has been able to induce long-lasting cellular and humoral immune responses. Taken together, these findings indicate that DNA-based vaccines are excellent candidates for translation into clinical trials for the development of practical AIDS vaccines. 
